# Supplementary material for: Health hazards related to using masks and/or personal protective equipment among physicians working in public hospitals in Dhaka: A cross-sectional study
Source: PLoS One. 2022 Sep 15;17(9):e0274169. doi: 10.1371/journal.pone.0274169 (PMC9477277; doi:10.1371/journal.pone.0274169)
Supplement: S2 Table — (DOCX) [file pone.0274169.s003.docx]

**Supplement 3: Univariate logistic regression analysis of the risk factors of respiratory distress, headache, facial disfigurement, and excessive sweating**

| **Trait** | | **Reference** | **P-value** | **OR** | **95%CI** |
| --- | --- | --- | --- | --- | --- |
| **Respiratory distress ^a^** | |  |  |  |  |
| **Age** | |  | 0.2 | 1.01 | 0.99-1.04 |
| **Sex** | | Male | 0.39 | 1.2 | 0.8-1.7 |
| Working in COVID dedicated Unit | | Yes | <0.001 | 2.1 | 1.4-3.1 |
| Physician position | | Supervisor | 0.008 | 0.65 | 0.47-0.89 |
| Duty pattern | | Roaster | 0.82 | 0.97 | 0.75-1.3 |
| Duty hour | | <8 hour | 0.003 | 0.5 | 0.34-0.8 |
| Evening practice | | yes | 0.35 | 0.84 | 0.58-1.2 |
| Practice duration | | >4 hour | 0.2 | 0.9 | 0.82-1.04 |
| Covid positive | | Yes | 0.28 | 1.2 | 0.89-1.5 |
| Covid symptoms | | Severe/critical | 0.04 | 1.2 | 1.01-1.4 |
| Protection used | | Mask only | 0.01 | 0.63 | 0.44-0.9 |
| Mask type | | Non-filtering | 0.14 | 0.87 | 0.72-1,05 |
| Frequency of mask use | | Infrequently | 0.89 | 0.97 | 0.63-1.5 |
| Time wear mask | | > 6 hour | 0.4 | 1.1 | 0.9-1.3 |
| Training on PPE | | Yes | 0.005 | 1.7 | 1.2-2.4 |
| Reuse of mask | | Yes | <0.001 | 1.9 | 1.4-2.9 |
| Asthma | | Yes | 0.001 | 2.4 | 1.4-3.9 |
| Diabetes | | Yes | 0.03 | 2.1 | 1.1-3.9 |
| hypertension | | Yes | 0.48 | 0.82 | 0.48-1.4 |
| IHD | | Yes | 0.79 | 1.5 | 0.09-23.2 |
| Obesity | | Yes | 0.001 | 2.6 | 1.5-4.8 |
| Personal stress level | | Low | 0.005 | 0.7 | 0.54-0.9 |
| Previous primary headache | | Yes | 0.82 | 1.04 | 0.7-1.5 |
| **Headache** | |  |  |  |  |
| **Age** | |  | 0.3 | 1.01 | 0.98-1.04 |
| **Sex** | | Male | 0.001 | 0.4 | 0.26-0.70 |
| Working in COVID dedicated Unit | | Yes | 0.001 | 2.1 | 1.4-3.1 |
| Physician position | | Supervisor | 0.9 | 1.01 | 0.7-1.4 |
| Duty pattern | | Roaster | 0.14 | 0.8 | 0.6-1.1 |
| Duty hour | | <8 hour | 0.6 | 1.1 | 0.7-1.8 |
| Evening practice | | yes | 0.4 | 1.2 | 0.8-1.8 |
| Practice duration | | >4 hour | 0.4 | 1.1 | 0.9-1.3 |
| Covid positive | | Yes | 0.1 | 1.3 | 0.9-1.7 |
| Covid symptoms | | Severe/critical | 0.06 | 1.2 | 0.9-1.4 |
| Protection used | | Mask only | 0.03 | 0.6 | 0.4-0.9 |
| Mask type | | Non-filtering | 0.3 | 0.9 | 0.7-1.1 |
| Frequency of mask use | | Infrequently | 0.3 | 1.4 | 0.8-2.4 |
| Time wear mask | | >6 hours | 0.4 | 0.9 | 0.8-1.1 |
| Training on PPE | | Yes | 0.07 | 1.4 | 0.9-2.2 |
| Reuse of mask | | Yes | 0.6 | 1.1 | 0.7-1.6 |
| Asthma | | Yes | 0.02 | 2.3 | 1.1-4.6 |
| Diabetes | | Yes | 0.06 | 2.5 | 0.9-6.6 |
| hypertension | | Yes | 0.09 | 1.8 | 0.9-6.6 |
| Obesity | | Yes | 0.04 | 0.5 | 0.3-0.9 |
| Personal stress level | | Low | 0.01 | 0.7 | 0.5-0.9 |
| Previous primary headache | | Yes | <0.001 | 4.5 | 2.5-8.1 |
| **Facial disfigurement** | |  |  |  |  |
| **Age** | |  | 0.9 | 1.01 | 0.9-1.02 |
| **Sex** | | Female | 0.001 | 2.2 | 1.3-3.5 |
| Working in COVID dedicated Unit | | Yes | 0.06 | 1.5 | 0.9-2.3 |
| Physician position | | Supervisor | 0.4 | 1.2 | 0.8-1.6 |
| Duty pattern | | Roaster | 0.2 | 0.8 | 0.5-1.1 |
| Duty hour | | <8 hour | 0.05 | 0.6 | 0.4-1.0 |
| Evening practice | | yes | 0.002 | 0.5 | 0.4-0.8 |
| Practice duration | | >4 hour | 0.01 | 0.8 | 0.7-0.9 |
| Covid positive | | Yes | 0.009 | 1.4 | 1.1-1.9 |
| Covid symptoms | | Severe/critical | 0.3 | 1.1 | 0.9-1.3 |
| Protection used | | Mask only | 0.001 | 0.5 | 0.3-0.8 |
| Mask type | | Non-filtering | 0.7 | 0.9 | 0.8-1.2 |
| Frequency of mask use | | Infrequently | 0.02 | 1.7 | 1.1-2.6 |
| Time wear mask | | > 6 hours | 0.02 | 1.3 | 1.01-1.6 |
| Training on PPE | | Absence | 0.2 | 1.3 | 0.9-1.9 |
| Reuse of mask | | Yes | <0.001 | 2.1 | 1.4-3.2 |
| Asthma | | Yes | 0.2 | 1.4 | 0.8-2.4 |
| Diabetes | | Yes | 0.9 | 1.1 | 0.5-2.2 |
| hypertension | | Yes | 0.9 | 0.9 | 0.5-1.8 |
| Obesity | | Yes | 0.9 | 1.02 | 0.6-1.9 |
| Personal stress level | | Low | 0.6 | 0.9 | 0.7-1.2 |
| Previous primary headache | | Yes | 0.5 | 0.9 | 0.6-1.3 |
| **Excessive sweating** | |  |  |  |  |
| **Age** | |  | 0.002 | 0.9 | 0.93-0.98 |
| **Sex** | | Female | 0.002 | 0.9 | 0.93-0.98 |
| Working in COVID dedicated Unit | | Yes | <0.001 | 2.6 | 1.6-3.9 |
| Physician position | | Supervisor | 0.03 | 1.4 | 1.02-2.01 |
| Duty pattern | | Roaster | 0.04 | 0.7 | 0.5-0.9 |
| Duty hour | | <8 hour | 0.01 | 0.5 | 0.3-0.9 |
| Evening practice | | yes | 0.1 | 0.9 | 0.3-0.7 |
| Practice duration | | >4 hour | 0.01 | 0.8 | 0.8-1.03 |
| COVID positivity | | Yes | <0.001 | 1.9 | 1.5-2.6 |
| Covid symptoms | | Severe/critical | 0.02 | 1.2 | 1.1-1.5 |
| Protection used | | Mask only | <0.001 | 0.2 | 0.2-0.4 |
| Mask type | | Non-filtering | 0.6 | 1.1 | 0.9-1.3 |
| Frequency of mask use | | Infrequently | 0.9 | 1.01 | 0.6=1.6 |
| Time wear mask | | >6 hours | 0.1 | 1.2 | 0.9-1.4 |
| Training on PPE | | Absence | <0.001 | 2.3 | 1.8-2.4 |
| Reuse of mask | | Yes | 0.008 | 1.7 | 1.1-2.5 |
| Asthma | | Yes | 0.7 | 0.9 | 0.5-1.6 |
| Diabetes | | Yes | 0.5 | 1.3 | 0.6-2.5 |
| hypertension | | Yes | 0.008 | 2.1 | 1.2-3.5 |
| Obesity | | Yes | 0.9 | 0.9 | 0.5-1.8 |
| Personal stress level | | Low | 0.5 | 1.1 | 0.8-1.4 |
| Previous primary headache | | Yes | 0.09 | 0.7 | 0.5-1.1 |
