# Supplementary material for: Health hazards related to using masks and/or personal protective equipment among physicians working in public hospitals in Dhaka: A cross-sectional study
Source: PLoS One. 2022 Sep 15;17(9):e0274169. doi: 10.1371/journal.pone.0274169 (PMC9477277; doi:10.1371/journal.pone.0274169)
Supplement: S1 File — (PDF) [file pone.0274169.s004.pdf]

Copy of health hazard PPE

Questions Responses Settings

Section 1 of 8

Health Hazards Related to Use of Masks and, or PPE among the Physicians of Bangladesh: A nation wide survey

Dear physician.
We are passing a very hard time in this COVID era. To make ourselves safe from this highly contagious disease we are using the PPE and masks. But it also has some hazards that we are experiencing every day. We planned to conduct a survey to know the extent and the types of hazards of masks or PPE.
This survey question will takes around 5-10 minutes to complete the answer. You will have to answer some questions regarding your experience of using mask/PPE and development of any hazards related to use of the masks and PPE. The terms and condition of this survey is explained below.

1.Confidentiality:
The information that we will collect from this research project will be kept confidential unless permitted by you. Information that will be collected from this study will only be used for research purpose. Your personal information will not be disclosed to anyone other than the investigators.

2. Right to refuse or withdraw:
You have all the right to refuse to participate in this study if you do not wish to do so. You may stop participating in this study at any time you wish.

3. Incentives:
You will not be provided any incentives to take part in this research.

give answer to any question or any portion of it if you need to do so.

5. Benefits:
You might not get direct benefit from this study. Your participation is likely to help us to acquire knowledge about the hazards and aware others. This will also help to find a solution of the hazards.

6.. Procedure of research:
If you agree, we will enroll you as a study participant and will adopt the following procedures for your participation-

i. We will take consent in the attached form

ii. you will have to answer some question attached herewith..

your participation will be highly appreciated

Thank you very much for your valuable time. Please feel free to contact in case of any query.

Thanking you.

Dr. Reaz Mahmud
Assistant professor, Neurology
Dhaka Medical college Hospital
Principal investigators of the present study

Email *

Valid email

This form is collecting emails. Change settings

After section 1 Continue to next section

Section 2 of 8

Consent of the participants

I am giving informed consent willingly to participate in the survey to be done by Dr. Reaz Mahmud. I agree to participate in the study voluntarily without any prejudice.

Do you agreed to participate in this survey? *

No

After section 2 Continue to next section

Section 3 of 8

Demography and general information of the of the participants

Description (optional)

What s your age?

Short answer text

Gender of the participants *

Male

Female

Do you work in a COVID dedicated hospital? *

Yes

No

- Professor
- Associate professor/Senior Consultant
- Assistant professor/Junior consultant
- Registrar
- Assistant registrar
- Medical officer
- Resident
- Other...

Pattern of your duty

- Roster
- Morning
- Supervising
- Administrative
- Other...

your usual duty hour

- 8 hour
- 12 hour
- 4-8 hour

Do you have evening practice

- Yes
- No

Average Duration of your practice

- 2 hour
- 3 hour
- 4 hour
- More than 4 hour

Have you been COVID-19 positive? *

- Yes
- No
- Maybe
- Multiple time

What was your presentation?

- Asymtomatic
- Mild
- Moderate

Critical

Your blood group

A positive

A negative

B positive

B negative

AB positive

AB negative

O positive

O negative

After section 3 Continue to next section

Section 4 of 8

About the use of PPE

Description (optional)

What type of protection you used?

Full PPE

- None
- Other...

Answer if you are not using any protection. Do you have any contraindication in using mask or PPE?

Multiple choice

Suggestions: Maybe

- yes
- No
- Add option or add "Other"

Required

Which Mask do you use mostly?

- N95
- KN95
- Surgical
- Gas respirator
- Home made mask
- Other...

How often you need to wear masks?

- weekly
- infrequent

Do you reuse the masks?

- Yes
- No
- Maybe

How much time you need to wear mask?

- >1 hour
- 1-2 hour
- 3-4 hour
- 4-6 hour
- 6-8 hour
- >8 hour

Do you have proper training on use of PPE?

- Yes
- No
- Maybe

Condition of your Doffing area

- ◯ Standard
- ◯ Average
- ◯ Below standard

After section 4 Continue to next section

Section 5 of 8

experience of using PPE OR MASK

Description (optional)

Do you experienced any major events after wearing mask or PPE

- ☐ syncopal attack
- ☐ severe respiratory distress
- ☐ severe chest pain
- ☐ Anaphylaxis
- ☐ None

How often you experience the serious adverse events?

- ◯ once

- every time
- infrequent

Do you experienced any minor events after wearing mask or PPE

- [ ] Headache
- [ ] Dizziness
- [ ] Irritation
- [ ] exertional dyspnoea
- [ ] chest pain
- [ ] excessive sweating
- [ ] panic attack
- [ ] disfigurement of the facies
- [ ] Other...

How often you experience the adverse events?

- infrequently
- often
- every time

After section 5 Continue to next section

Functional impact of the adverse events

Description (optional)

Please answer if you experience Dyspnoea

- Breathlessness on strenous exercise
- Breathless on hurring or walking uphill
- Need to slowdown the pace of the walk due to breathlessness
- Stop walking after few minute due to brathlessness
- Breathlessness in routine work, like dressing, talking etc

Please answer if you have headache

Short answer text

When you have headaches, how often is the pain severe?

- Never
- Rarely
- Sometimes
- Veryoften
- Always

How often do headaches limit your ability to do usual daily activities including household work, work, school, or social activities?

- Never
- rarely
- Sometimes
- Very Often
- Always

When you have a headache, how often do you wish you could lie down?

- Never
- Rarely
- Sometimes
- Often
- Always

I n the past 4 weeks, how often have you felt too tired to do work or daily activities because of your headaches?

- Never
- Rarely
- Sometimes
- Often

In the past 4 weeks, how often have you felt fed up or irritated because of your headaches?

- Never
- Occasionally
- Sometimes
- Often
- Always

In the past 4 weeks, how often did headaches limit your ability to concentrate on work or daily activities?”

- Never
- Occasionally
- Sometimes
- Often
- Always

After section 6 Continue to next section

Section 7 of 8

THANKS FOR YOUR PARTICIPATION

Description (optional)

After section 7 Continue to next section

Section 8 of 8

Untitled Section

Description (optional)
