# Supplementary material for: Health hazards related to using masks and/or personal protective equipment among physicians working in public hospitals in Dhaka: A cross-sectional study
Source: PLoS One. 2022 Sep 15;17(9):e0274169. doi: 10.1371/journal.pone.0274169 (PMC9477277; doi:10.1371/journal.pone.0274169)
Supplement: Attachment — Submitted filename: comments.docx [file pone.0274169.s008.docx]

**Manuscript Title: Health hazards related to using masks and, or personal protective equipment among the physicians working in different public hospitals in Dhaka: A cross-sectional survey**

1. **Title**: it is researchable, interesting and targeted. The manuscript had presented **Health hazards related to using masks and, or personal protective equipment among the physicians working in public hospitals**. It can be a valuable scientific literature if published after incorporation of the comments raised.

2. **Abstract**:
-

**3. Introduction**

**-**

**4. Methods**

- Better to state about the brief background of your study area, from how many hospitals the physicians recruited.
- Why you conduct only on physicians, what about other health care workers like Nurses, pharmacists, midwiferies, Anesthesia…?
- If you conceptual framework it is better to show the relationship of predictor and outcome variables in the form of conceptual framework.
- Better to clarify about your sample size determination, I think you used census method, so what is the advantage of using single population proportion formula?
- What types of technique you used to keep/control your data quality? Since data quality is the corner stone for mixed research approach.
- I haven’t seen about your tool/questionnaire or checklist you used to collect your data; therefore, please try to state about the checklist you used and where you adopt or adapt the checklist or questionnaire. .

**5. Result**

- No need to state repeat similar concepts in different data presentation form. Therefore, it is better to state either in table or graph with brief description, or in text form….
- Your logistic regression model is not clear?
  - You only conducted bivariable logistic regression which is not enough and didn’t consider the confounders, and your odds ratio is not clear whether it is AOR or COR.
    - If your odds ratio (OR) is Adjusted Odds Ratio (AOR), where is your COR and what was the criteria to pass your variables from bivariable to multi variable logistic regression?
    - If your odds ratio (OR) is COR you need to conduct multivariable regression model for better decision since COR is didn’t consider other confounding variables.
- If you refine the above things, your study would be valuable. Therefore, try to focus on your logistic regression model.

**Decision: Major revision**
