# Supplementary material for: Health hazards related to using masks and/or personal protective equipment among physicians working in public hospitals in Dhaka: A cross-sectional study
Source: PLoS One. 2022 Sep 15;17(9):e0274169. doi: 10.1371/journal.pone.0274169 (PMC9477277; doi:10.1371/journal.pone.0274169)
Supplement: Attachment — Submitted filename: Response to Reviewers.docx [file pone.0274169.s010.docx]

**A rebuttal letter**

To

Editor in chief,

PLOS ONE

**Subject: In response to review of the manuscript entitled “Health hazards related to using masks and/or personal protective equipment among physicians working in public hospitals in Dhaka: a cross-sectional study”.**

Dear Sir,

Thank you for reviewing my manuscript. I have tried to address each point raised by the academic editor and the reviewers.

**Response to Academic editor**

1. Why you used the heading *“Methods and Materials”?* Is there a material that used for this study? if not, please omit the “materials”.

Response: Thank you for the note. There is no material used in this study. It was a mistake. I have omitted materials. (line 150)

2. The methods will be better if structured in this way; Study Area and Period, Study Design, source population, study population, Eligibility criteria, Sample Size Determination, Sampling Technique and Procedures, Study Variables, Operational Definitions, Data Collection Instrument, Data Collection Procedures, Data Quality Control, and Data Processing and Analysis. Specially, this section should be critically addressed!

Response: Thanks for the advice. I have revised the methods as per your instructions.

(Line-154,160,169,172, 178, 182, 212,253,259, 265,277, 288)

3. The authors should clearly explain their Eligibility criteria as; inclusion and exclusion criteria.

Response: mentioned in the specific section (line 172-177)

4. The authors must operationalize their outcome variables. How did they measure their outcome variables?

Response: the definition of the outcome variables was added in the operational definition section and Measurement of dyspnea was made with MRC score and headache with HIT-6 score questionnaire.

(Line: 199-211)

5. Concerning to the sample size calculation for this study, the authors explained as …. So the estimated sample size was 384. We included a total of 506 respondents in this study” why you didn’t used the calculated sample size? This needs a justification.

Response: We were not sure how many of the approached physicians would respond. So, we approached to 1122 physicians and 506 responses were ultimately included in the study. As this line is creating confusion, we deleted this line.

6. How did they get the study subjects? They have to clearly address their sampling technique?

Response: added in the corresponding section (line 182-185)

7. Since you conducted the data collection with online means, how did you keep the data quality for this study?

Response: added in the corresponding section (line-265-276)

8. Study instrument: have you developed, adapted or adopted the questionnaires? You have to clearly explain about the questionnaires you used because this is crucial. You have to report the reliability analysis finding for your questionnaires.

Response: we developed the questionnaire. We made the reliability with Cronbach’s alpha, which was 0.7. and validity test with Pearson correlation. Added in the text. (line-267-279)

9. Add the response rate.

Response: We accessed a total of 1122 doctors; among them, 534 doctors responded. Response rate (47.6%). Added in the text (line 292-293)

10. **Discussion:** discuss by using the scientific reasoning.

**Response:** some revision made as per your instruction

11. **Conclusion:** avoid numbering. Make it a paragraph. Focus on your main findings, clinical implications, recommendations etc.

**Response:** Corrected in the text as per your instruction

**Response to the reviewer:**

Thank you for reviewing the article. I have tried to address all the points raised in your review comments.

Reviewer #1: Manuscript Title: Health hazards related to using masks and, or personal protective equipment among the physicians working in different public hospitals in Dhaka: A cross-sectional survey
1. Title: it is researchable, interesting and targeted. The manuscript had presented Health hazards related to using masks and, or personal protective equipment among the physicians working in public hospitals. It can be a valuable scientific literature if published after incorporation of the comments raised.
2. Abstract:
-
3. Introduction
-
4. Methods
♣ Better to state about the brief background of your study area, from how many hospitals the physicians recruited.

Response: Stated (line-157-159)
♣ Why you conduct only on physicians, what about other health care workers like Nurses, pharmacists, midwiferies, Anesthesia…?

Response: We made the case record form in English and we used lot of medical terms. In Bangladesh we thought it would be difficult for the Nurses, pharmacist, midwiferies to understand every aspect of the case record form.
♣ If you conceptual framework it is better to show the relationship of predictor and outcome variables in the form of conceptual framework.

Response: this study is not a conceptual frame work
♣ Better to clarify about your sample size determination, I think you used census method, so what is the advantage of using single population proportion formula?

Response: we included only the physicians. So, we used single population proportion formula
♣ What types of technique you used to keep/control your data quality? Since data quality is the corner stone for mixed research approach.

Response: Thanks for your concern. I have revised the writing and explained it in line-265-276.
♣ I haven’t seen about your tool/questionnaire or checklist you used to collect your data; therefore, please try to state about the checklist you used and where you adopt or adapt the checklist or questionnaire. .

Response: added in the supplement-1


5. Result
♣ No need to state repeat similar concepts in different data presentation form. Therefore, it is better to state either in table or graph with brief description, or in text form….

Response: Thank you for your advice. I have corrected as much as possible.
♣ Your logistic regression model is not clear?
o You only conducted bivariable logistic regression which is not enough and didn’t consider the confounders, and your odds ratio is not clear whether it is AOR or COR.
♣ If your odds ratio (OR) is Adjusted Odds Ratio (AOR), where is your COR and what was the criteria to pass your variables from bivariable to multi variable logistic regression?
♣ If your odds ratio (OR) is COR you need to conduct multivariable regression model for better decision since COR is didn’t consider other confounding variables.

Response: It was adjusted odd ratio. We used forward conditional methods in the regression model. Crude odd ration was added in the supplement 3. (line 342-344)
¬ If you refine the above things, your study would be valuable. Therefore, try to focus on your logistic regression model.

**Reviewer #2: Reviewer comments**

Manuscript Number: PONE-D-21-25497

Title "Health hazards related to using masks and, or personal protective equipment among the physicians working in different public hospitals in Dhaka: A cross-sectional study".

Generally speaking:
Thank you for providing me the opportunity to review this manuscript that raises important issues about health hazards related to using masks and/or personal protective equipment in one of the developing countries.

Comment 1:
1. ABSTRACT:
a) Results:
• The number of samples should be mentioned in the methodology of the abstract.

Response: Added (line-80)
• (35.4[7.7]) these numbers should be clarified.

Response: It was mean (SD) age, corrected (Line-83)
• Furthermore, the feminine female gender and reusing masks for an extended period were the risk factors for facial disfigurement.

Response: Rewritten as “Furthermore, the female gender, reusing masks for an extended period (> 6 hours) were the risk factors for facial disfigurement.”

Comment 2:
2. INTRODUCTION:
a) The text of the introduction does not seem to have coherence and integrity. It should be concise and targeted to the aim.
b) Global/ Regional/ Bangladesh prevalence of health hazards related to using of personal protective equipment among health workers should be mentioned. The current situation of other developing and developed countries should also be added.

Response: Thank you for your advice. I have rewritten this section, tried to be concise and targeted as per your advice. But we found very few comprehensive studies in this regard. So current situation in the developed and developing countries cannot be added. I believe it would be better if we could add it.
c) Risk factors associated with health hazards related to using of personal protective equipment should be clearly stated.

Response: Added in the introduction to some extent, according to other studies. (line-137-139)

d) Explaining why this topic was chosen for analysis in this article is not well written. The benefits of conducting the study to the community should be explained.

Response: Rewritten. Line-146-149

Comment 3:
3. METHODS:
Generally, the information mentioned under the methods are too long with redundancy and diffusion and should be divided into several sub-sections as follow:
a) Type of the study
b) Study setting
c) Study Participants: The characteristics of the study participants should be mentioned as inclusion criteria and exclusion criteria (if any)
d) Sample size
e) Study tools:
• There was no clear mention of the questionnaire used. Was it newly developed by the authors (if so, include the reference)? Was it piloted to assess its internal consistencies? Was it validated?
• It is advisable to include the questions as per each domain in the methodology, how to score the questionnaire, specify the cut points in the questionnaire, as well as how long did it take to complete each questionnaire?
f) Data management analysis
g) Ethical considerations

Response: rewritten as per instruction (line-154,160,169,172, 178, 182, 212,253,259, 265,277, 288)

Comment 4:
4. RESULTS:
1) In line 237, I don't agree with the term ''mostly'' when describing those worked in COVID-19 dedicated units, they were about two thirds and not the most of study population. Again, in line 238, the term ''most'' for describing the respondents worked on the roster is not true, they were three quarters and not the most of study population. In line 280, the term ''most'' was repeated twice while the percentages were 52% and 39%. It is advisable to revise the rest of the result comments.

Response: Thank you for your advice I have corrected these. (line- 299, 300, 301 etc)
2) How does increasing personal stress level increased the chance of dyspnea with any severity, while OR is 0.7.

Response: corrected (Line-348-349)
3) In line 284, it is table 2 not table 3.

Response: Corrected
4) Assessment of Relative Risk in table 1 should be explained.

Response: I have rewritten it and explained in 309-323

Comment 5:
5. DISCUSSION:
a) Should give reasons for the risk factors. The manuscript could be greatly strengthened if the authors could provide highlight on the risk factors in other developing and developed countries with similar context.

Response: Thank you for your advice. I have rewrite tis section, tried to focused on your concern Limited literature was found even after extensive search to compare.
b) Compare the findings of the study with other findings and state the reasons for the strengths and weaknesses in each section.

Response: Tried to address as far as possible. We found a few study to compare.
a) Line 356: “Headache prevalence is similar to other studies”. It is advisable to give the prevalence of headache and other health hazards which was not mentioned, as well as the prevalence of the risk factors.

Response: Corrected (line 419, 440,
b) Line 364: regular psychological assessment and counseling for managing the stress

Response: Corrected
c) The sentence “It was a cross-sectional study” was repeated many times. e.g, it was repeated two times in the last paragraph of the discussion.

Response: Corrected

Comment 6:
6. CONCLUSION:
It is unclear. It should be specific and based on the findings of the study.

Response: The writing of the conclusion was revised.

Comment 7:
7. STRENGTHS AND LIMITATIONS:
a) Please analyze the strengths of the study.
b) Last paragraph of discussion is the limitations of the study

Response: Added in the text

Comment 8:
8. REFERENCES:
a) Please revise the Harvard Vancouver method.
b) All references should be written in the same way.

Response: I have revised as per your instruction

Comment 9:
English needs to be revised.

Response: The manuscript has been revised by Editage for English language

***I hope I have tried my level best to address all of your point raised during the review process. Please consider my manuscript for publication in PLOS ONE.***

***Thanks***

***Dr. Reaz Mahmud***

***Assistant professor Neurology***

***Dhaka Medical College***
