# Supplementary material for: Health hazards related to using masks and/or personal protective equipment among physicians working in public hospitals in Dhaka: A cross-sectional study
Source: PLoS One. 2022 Sep 15;17(9):e0274169. doi: 10.1371/journal.pone.0274169 (PMC9477277; doi:10.1371/journal.pone.0274169)
Supplement: Attachment — Submitted filename: Response to Reviewers.docx [file pone.0274169.s012.docx]

**A rebuttal letter**

To

Editor in chief,

PLOS ONE

**Subject: In response to review of the manuscript entitled “Health hazards related to using masks and/or personal protective equipment among physicians working in public hospitals in Dhaka: a cross-sectional study”.**

Dear Sir,

Thank you for reviewing my manuscript. I have tried to address each point raised by the academic editor and the reviewers.

**Response to Academic editor:**

Thank you for giving me the opportunity to revise the article.

**Response to the reviewer:**

Thank you for reviewing the article. I have tried to address all the points raised in your review comments.

**Reviewer #2:**

Thank you for providing me the opportunity to review this manuscript that raises important issues about health hazards related to using masks and/or personal protective equipment in one of the developing countries.

It seems that all corrections were done.

Response: Thank you for accepting the corrections

**Reviewer #3:**

The authors of this manuscript have improved upon its quality substantially based on the comments from the first review. However, the authors should address the comments raised below:
Line 121: Change these words "As frontliners of this pandemic," to read "As frontliners in the control of this pandemic,"

Response: Thank you for the correction. I have corrected as per your advice.
Line 124: Are there updated/more recent figures on the mortality of HCWs due to COVID-19? Since this study focused on physicians only, are there specific records for physicians only?

Response:

I have searched extensively for updated/recent figures, but failed to find updated figures on physicians only.

I have found an estimate of WHO, So I added the following statement in the manuscript “Reported death to WHO COVID-19 surveillance in health care workers due to COVID -19, from January 2020–May 2021 was only 6643. Still, a population-based estimate revealed that 115 493 health and care workers could have died from COVID-19 during that period”

.Line 154: What are the reasons for selecting these public hospitals? How many public hospitals are in Bangladesh? Are these hospitals representative enough? A map of Bangladesh showing the sampled hospitals would be a good fit.

Response: There are 3,976 healthcare facilities in the public sector in Bangladesh; It includes union subcenters. Among them, 600 facilities were dedicated to treating COVID-19 patients nationwide. We did the study in Dhaka city, the capital of Bangladesh. In Dhaka city, nine large public hospitals were involved in COVID treatment. Among them, we choose these five hospitals purposively. These hospitals are representative enough of Dhaka city. We did not include other health facilities nationwide, as health facilities outside Dhaka are not homogenous, and there is a communication problem.

To avoid redundancy, we did not mention all this information in the manuscript. We corrected the statement below” Among nine large COVID-19 dedicated hospitals in Dhaka city, we purposively selected these five hospitals.” A Map of Dhaka showing this hospital has been attached as supplements.
Lines 172-177: As part of the eligibility criteria, the authors should indicate within the manuscript the reason why other HCWs were excluded from the study.

Response: added in 195-197.
Lines 178-181: Authors should include a reference for using the stated formula in calculating the sample size. Furthermore, the authors must include a justification within the manuscript as to the reason why respondents well-above the calculated sample size were recruited into the study.

Response: Reference added line-199, Justification of recruiting repondents well above the calculated sample size added, line-201-203.

Line 182: I believe the authors have used a non-probability sampling technique "Purposive" since physicians were targeted. It won't be out of place to indicate that a Purposive sampling technique was used.

Response: we deleted the line
Line 284: Why have the authors considered the use of Relative risk and not odds ratio in this cross-sectional study?

Response: Thanks for pointing this. It was a mistake, so we corrected it. Line 318.
Table 1: Authors should include among the keys below the table the full terms of all abbreviations used.
Table 3: Authors should include among the keys below the table the full terms of all abbreviations used.

Response: Added in the manuscript
The authors should include a paragraph just before the conclusion stating clearly the limitations associated with this study and how these limitations affect the generalizability of their findings

Response: added in 604-621 lines.

**Journal Requirements:**

**I have reviewed the references and found one reference might be retracted one, so deleted that reference, number 32.**

***“***Warren DW, Mayo R, Zajac DJ, Rochet AH. Dyspnea following experimentally induced increased nasal airway resistance. Cleft Palate Craniofac J. 1996 May;33(3):231-5. doi: 10.1597/1545-1569_1996_033_0231_dfeiin_2.3.co_2. PMID: 8734724.”

I have added two more references as per requirement of the reviewers

No-8: World Health Organization‎. The impact of COVID-19 on health and care workers: a closer look at deaths. World Health Organization. 2021, Accessed on 10.08.2022. <https://apps.who.int/iris/handle/10665/345300>.

No-16: Daniel WW, editor. 7th ed. New York: John Wiley & Sons; 1999. Biostatistics: a foundation for analysis in the health sciences.

***I hope I have tried my level best to address all of your point raised during the review process. Please consider my manuscript for publication in PLOS ONE.***

***Thanks***

***Dr. Reaz Mahmud***

***Assistant professor Neurology***

***Dhaka Medical College***
