# Supplementary material for: Health hazards related to using masks and/or personal protective equipment among physicians working in public hospitals in Dhaka: A cross-sectional study
Source: PLoS One. 2022 Sep 15;17(9):e0274169. doi: 10.1371/journal.pone.0274169 (PMC9477277; doi:10.1371/journal.pone.0274169)
Supplement: Attachment — Submitted filename: Reviewer comments PONE-D-21-25497.docx [file pone.0274169.s009.docx]

Reviewer comments

Manuscript Number: PONE-D-21-25497

Title "Health hazards related to using masks and, or personal protective equipment among the physicians working in different public hospitals in Dhaka: A cross-sectional study".

**Generally speaking:**
Thank you for providing me the opportunity to review this manuscript that raises important issues about health hazards related to using masks and/or personal protective equipment in one of the developing countries.

**Comment 1:**
*1. ABSTRACT:*

a) Results:

- The number of samples should be mentioned in the methodology of the abstract.
- (35.4[7.7]) these numbers should be clarified.
- Furthermore, ~~the feminine~~ female gender and reusing masks for an extended period were the risk factors for facial disfigurement.

**Comment 2:**
*2. INTRODUCTION:*

a) The text of the introduction does not seem to have coherence and integrity. It should be concise and targeted to the aim.
b) Global/ Regional/ Bangladesh prevalence of health hazards related to using of personal protective equipment among health workers should be mentioned. The current situation of other developing and developed countries should also be added.
c) Risk factors associated with health hazards related to using of personal protective equipment should be clearly stated.
d) Explaining why this topic was chosen for analysis in this article is not well written. The benefits of conducting the study to the community should be explained.

**Comment 3:**
*3. METHODS:*

Generally, the information mentioned under the methods are too long with redundancy and diffusion and should be divided into several sub-sections as follow:

a) Type of the study
b) Study setting
c) Study Participants: The characteristics of the study participants should be mentioned as inclusion criteria and exclusion criteria (if any)
d) Sample size
e) Study tools:

- There was no clear mention of the questionnaire used. Was it newly developed by the authors (if so, include the reference)? Was it piloted to assess its internal consistencies? Was it validated?
- It is advisable to include the questions as per each domain in the methodology, how to score the questionnaire, specify the cut points in the questionnaire, as well as how long did it take to complete each questionnaire?

f) Data management analysis
g) Ethical considerations

**Comment 4:**
*4. RESULTS:*

1) In line 237, I don't agree with the term ''mostly'' when describing those worked in COVID-19 dedicated units, they were about two thirds and not the most of study population. Again, in line 238, the term ''most'' for describing the respondents worked on the roster is not true, they were three quarters and not the most of study population. In line 280, the term ''most'' was repeated twice while the percentages were 52% and 39%. **It is advisable to revise the rest of the result comments.**
2) How does increasing personal stress level increased the chance of dyspnea with any severity, while OR is 0.7.
3) In line 284, it is table 2 not table 3.
4) Assessment of Relative Risk in table 1 should be explained.

**Comment 5:**

*5. DISCUSSION:*

a) Should give reasons for the risk factors. The manuscript could be greatly strengthened if the authors could provide highlight on the risk factors in other developing and developed countries with similar context.
b) Compare the findings of the study with other findings and state the reasons for the strengths and weaknesses in each section.
a) Line 356: “Headache prevalence is similar to other studies”. It is advisable to give the prevalence of headache and other health hazards which was not mentioned, as well as the prevalence of the risk factors.
b) Line 364: regular psychological assessment and counseling for managing the stress
c) The sentence “It was a cross-sectional study” was repeated many times. e.g, it was repeated two times in the last paragraph of the discussion.

**Comment 6:**

*6. CONCLUSION:*

It is unclear. It should be specific and based on the findings of the study.

**Comment 7:**

*7. STRENGTHS AND LIMITATIONS:*

a) Please analyze the strengths of the study.
b) Last paragraph of discussion is the limitations of the study

**Comment 8:**

*8. REFERENCES:*

a) Please revise the Harvard Vancouver method.
b) All references should be written in the same way.

**Comment 9:**

English needs to be revised.
