# Supplementary material for: Health hazards related to using masks and/or personal protective equipment among physicians working in public hospitals in Dhaka: A cross-sectional study
Source: PLoS One. 2022 Sep 15;17(9):e0274169. doi: 10.1371/journal.pone.0274169 (PMC9477277; doi:10.1371/journal.pone.0274169)
Supplement: Attachment — Submitted filename: Reviewer comments PONE-D-21-25497_R1.docx [file pone.0274169.s011.docx]

Reviewer comments

Manuscript Number: PONE-D-22-03316R1

Title "Health hazards related to using masks and, or personal protective equipment among the physicians working in different public hospitals in Dhaka: A cross-sectional study".

Thank you for providing me the opportunity to review this manuscript that raises important issues about health hazards related to using masks and/or personal protective equipment in one of the developing countries.

It seems that all corrections were done.
